# Lymphatic Microvascular Density, Lymphatic Invasion and Endothelial Cell Proliferation as Prognostic Biomarkers in Cutaneous Melanoma: A Systematic Review and Evidence Map

**DOI:** 10.3390/cancers18203412

**Published:** 2026-10-21

**Authors:** Victor Cristian Dumitrascu, Amalia Raluca Ceaușu, Mihai Mitulețu, Călin Muntean, Roxana Popescu, Gabi Dumitrescu, Pusa Nela Gaje, Marius Raica, Cristian Sebastian Vlad

**Affiliations:** 1 Doctoral School, Faculty of Medicine, “Victor Babeș” University of Medicine and Pharmacy, 2 Eftimie Murgu Square, 300041 Timișoara, Romania; cristian.dumitrascu@umft.ro; 2 Department of Microscopic Morphology, Histology, Angiogenesis Research Center, “Victor Babeș” University of Medicine and Pharmacy, 2 Eftimie Murgu Square, 300041 Timișoara, Romania; ra.ceausu@umft.ro (A.R.C.); raica@umft.ro (M.R.); 3 ANAPATMOL Research Center, “Victor Babeș” University of Medicine and Pharmacy, 2 Eftimie Murgu Square, 300041 Timișoara, Romania; popescu.roxana@umft.ro (R.P.); gdumitrescu@animalsci-tm.ro (G.D.); 4 Department of Microscopic Morphology, Discipline of Cellular and Molecular Biology, “Victor Babeș” University of Medicine and Pharmacy, 2 Eftimie Murgu Square, 300041 Timișoara, Romania; 5 Department of Informatics and Medical Biostatistics, “Victor Babeș” University of Medicine and Pharmacy, 2 Eftimie Murgu Square, 300041 Timișoara, Romania; 6 Faculty of Bioengineering of Animal Resources, University of Life Sciences “King Mihai I” from Timișoara, 119 Calea Aradului, 300645 Timișoara, Romania; 7 Department of Biochemistry and Pharmacology, Faculty of Medicine, “Victor Babeș” University of Medicine and Pharmacy, 2 Eftimie Murgu Square, 300041 Timișoara, Romania; vlad.cristian@umft.ro

**Keywords:** cutaneous melanoma, lymphangiogenesis, lymphatic vessel density, lymphatic invasion, podoplanin, D2-40, Ki-67, microvascular density, sentinel lymph node, systematic review, evidence map

## Abstract

**Background/Objectives:** Cutaneous melanoma spreads preferentially through the lymphatic system, and sentinel lymph node status is among the strongest prognostic factors. Whether tumour angiogenesis and lymphangiogenesis add prognostic information beyond conventional histopathology has been examined in many immunohistochemical studies, with heterogeneous results. We conducted a systematic review, reported according to PRISMA 2020 and not registered, of blood microvascular density (MVD), lymphatic vessel density (LVD) in the intratumoral and peritumoral compartments, proliferating (Ki-67-positive) lymphatic vessels and lymphatic invasion (LI) as prognostic markers in primary cutaneous melanoma. **Methods:** PubMed/MEDLINE was searched to 10 September 2026, supplemented by a Web of Science export and by citation tracking of the reference meta-analysis by Pastushenko et al. (2014). Twenty-one cutaneous study units (25 reports) and four ocular studies providing indirect evidence were included, and risk of bias was rated with the QUIPS tool. **Results:** In the reference meta-analysis, peritumoral LVD and LI, but not MVD or intratumoral LVD, were associated with metastasis. The newer cohorts confirmed this pattern and provided adjusted effect sizes: peritumoral LVD predicted disease-free (HR 2.14) and melanoma-specific survival (HR of 2.74; 95% CI 1.31 to 5.73), immunohistochemically detected LI predicted sentinel node metastasis (33% vs. 11% positive nodes) and overall survival, and a Ki-67-positive lymphatic endothelium predicted melanoma-specific mortality (adjusted HR of 2.03; 95% CI 1.33 to 3.10) in 264 patients. Blood MVD and blood endothelial proliferation were not prognostic. Heterogeneity of markers, counting methods and effect measures precluded re-pooling. **Conclusions:** Peritumoral LVD, LI and proliferating lymphatics are promising adjunct biomarkers for selecting patients for sentinel node biopsy, adjuvant therapy and closer follow-up, pending standardised, digital-pathology-based quantification.

## Simple Summary

Melanoma is a skin cancer that often spreads first through lymphatic vessels, the thin channels that drain fluid from the skin to the lymph nodes. Pathologists can see these vessels in a tumour sample with a stain called D2-40, and they can tell which vessels are growing with a second stain, Ki-67, which marks dividing cells. Three measurements are of interest. Lymphatic vessel density (LVD) is the number of lymphatic vessels in a defined area, either inside the tumour or at its edge. Lymphatic invasion (LI) means that tumour cells are found inside a lymphatic vessel. Endothelial proliferation means that the cells lining the vessels are dividing. We reviewed 26 studies to find out whether these measurements predict spread to the lymph nodes and survival. A high density of lymphatic vessels at the tumour edge, tumour cells inside lymphatic vessels and dividing lymphatic vessels were each associated with spread and with poorer survival. The number of blood vessels was not. These measurements are inexpensive and could help to decide which patients need a sentinel lymph node biopsy or closer follow-up, but they must first be measured in the same way in every laboratory.

## 1. Introduction

Cutaneous melanoma accounts for a small fraction of skin cancers but for most skin cancer deaths because of its propensity for early metastatic spread. An estimated 325,000 new cases and 57,000 deaths occurred worldwide in 2020, and the burden is projected to rise by about half by 2040 [1,2,3]. Prognosis depends strongly on stage. In the eighth edition of the American Joint Committee on Cancer (AJCC) staging system, five-year melanoma-specific survival falls from 99% in stage IA to 82% in stage IIC and 32% in stage IIID, and stage is determined mainly by Breslow thickness, ulceration and nodal status [4,5]. After curative surgery, recurrence remains common in the higher stages. In the placebo arms of two recent adjuvant trials in resected stage IIB and IIC melanoma, 17% to 21% of patients had relapsed within the first year [6,7], and in resected stage III disease, the one-year recurrence-free survival without adjuvant treatment was 61% [8]. In the metastatic setting, which, until recently, carried a median survival of well under two years, combined immune checkpoint blockade now achieves a median overall survival of about six years, with 37% of patients alive at ten years, compared with 20 months with ipilimumab alone [9]. These figures explain why risk stratification matters at every stage of the disease: it decides who should be offered sentinel lymph node biopsy, adjuvant therapy or intensified follow-up and who can safely be spared them.

Breslow thickness, ulceration, mitotic rate and nodal status do not fully capture the biological aggressiveness of an individual tumour, so additional biomarkers have been sought within the tumour microenvironment—in particular, in its vascular and stromal components [10,11,12]. Tumour angiogenesis is an established feature of melanoma progression, and blood microvascular density (MVD), most often assessed by the Weidner hot-spot method, has been proposed as a prognostic indicator [13,14,15,16]. The lymphatic system, however, is the principal early route of melanoma dissemination. Tumour lymphangiogenesis, the formation of new lymphatic vessels driven chiefly by VEGF-C and VEGF-D signalling through VEGFR-3, has therefore attracted increasing attention, and lymphatic vessels are now recognised as active regulators of tumour immunity rather than passive conduits [17,18,19,20,21].

Three tissue measurements are at the centre of this review, and the terms used for them need definition. Lymphatic vessel density (LVD) is the number of lymphatic vessels per unit area, identified specifically with monoclonal antibody D2-40 against podoplanin or with LYVE-1 [22,23]. It is measured in two compartments: the intratumoral compartment, within the tumour mass, and the peritumoral compartment, in the stroma immediately outside the invasive front. The ratio between the two is the peripheral-to-central gradient. Lymphatic invasion (LI) is the presence of tumour cells within the lumen of a lymphatic vessel, best detected by dual immunohistochemistry for a lymphatic marker and a melanocytic marker. Endothelial cell proliferation is resolved by co-staining D2-40 with proliferation marker Ki-67, which separates actively growing lymphatic vessels from the pre-existing, quiescent network; we call a study proliferation-resolved when it reports this fraction separately. Despite a large number of immunohistochemical studies, the prognostic value of these parameters has been inconsistent, differing by compartment and by the marker used [24,25,26,27].

A systematic review and meta-analysis by Pastushenko and colleagues, published in 2014, synthesised the literature available at that time and concluded that peritumoral LVD and LI have prognostic value in melanoma, whereas MVD and intratumoral LVD do not [24]. Four developments since then justify re-examining the question. First, more than fifteen new cohorts have been published—among, them two large series that co-stained D2-40 with Ki-67 and several that applied dual staining to detect LI. Second, quantification has moved from manual hot-spot counts towards Chalkley counting, computer-assisted morphometry and multiplex imaging, which changes both the precision and the comparability of the measurements. Third, lymphatic vessels have been shown to shape antitumour immunity so that a high LVD may mean something different in the era of adjuvant immunotherapy. Fourth, the clinical question itself has changed: with adjuvant checkpoint inhibitors now approved from stage IIB and completion lymphadenectomy largely abandoned, the value of a tissue biomarker lies in refining the selection of patients for sentinel lymph node biopsy, adjuvant treatment and follow-up. No systematic review has brought this evidence together, and the pooled analysis has not been updated. The aims of the present work were therefore to systematically review the evidence on MVD, LVD by compartment, proliferating lymphatic vessels and LI as prognostic biomarkers in primary cutaneous melanoma; to appraise the risk of bias of the available cohorts; and to construct an evidence map, graded by explicit criteria, that identifies the biomarker, compartment and outcome combinations best supported by current data.

## 2. Materials and Methods

### 2.1. Protocol, Registration and Reporting

This review was performed in accordance with the PRISMA (Preferred Reporting Items for Systematic Reviews and Meta-Analyses) 2020 guidelines and has not been registered [28]. The completed PRISMA 2020 checklist is provided as Supplementary Table S1. The review follows the methodological guidance for systematic reviews of prognostic factor studies [29], and the quantitative synthesis is anchored on the reference meta-analysis by Pastushenko et al. [24], which was not re-pooled for the reasons given in Section 2.6.

### 2.2. Eligibility Criteria

Eligibility was framed in Population, Exposure, Comparator and Outcome (PECO) terms (Table 1). Eligible studies enrolled patients with histopathologically confirmed primary cutaneous melanoma; assessed blood MVD, LVD, proliferating lymphatic vessels or LI by immunohistochemistry or digital pathology, with spatial information (intratumoral, invasive front or peritumoral) where available; compared higher versus lower density, metastatic versus non-metastatic tumours, or peritumoral versus intratumoral compartments; and reported at least one clinicopathological or prognostic outcome—namely, sentinel lymph node (SLN); regional or distant metastasis; recurrence or disease-free survival; melanoma-specific or overall survival; or an association with an established prognostic variable such as thickness, ulceration or stage. Cohort, case–control and analytical cross-sectional studies with at least 10 human cases were eligible. Studies of ocular, conjunctival and mucosal melanoma did not meet the population criterion; those reporting proliferating lymphatic vessels were retained as indirect evidence, tabulated separately and not used for grading of the cutaneous evidence map. Cell lines, animal models, case reports, reviews and studies without tissue-level vascular quantification were excluded. Unpublished data from the authors’ own institutions were not eligible in order to avoid a conflict of interest in the evidence base; the authors’ published cohort on blood microvessels and mast cells [30] was screened against the same criteria as any other report.

### 2.3. Information Sources and Search Strategy

Four sources were used. First, PubMed/MEDLINE was searched from inception to 22 July 2026 with a core strategy that combined melanoma terms with vascular and lymphatic terms and, in a sensitised version, omitted the spatial block (Table 2). Second, a Web of Science Core Collection export of 50 recent records retrieved with the same concept blocks was screened. Third, an update search of PubMed/MEDLINE restricted to 2013 onwards, the period not covered by the reference meta-analysis, was run on 10 September 2026 with five overlapping query strings covering lymphatic vessel density and lymphangiogenesis; lymphatic and lymphovascular invasion; Ki-67 in combination with lymphatic markers; VEGF-C; VEGF-D and VEGFR-3 with outcome terms; and compartment terms such as peritumoral, intratumoral and invasive front. Fourth, forward citation tracking of the reference meta-analysis was performed in PubMed on 14 October 2026, and the reference lists of the included reports were screened. Embase, Scopus and the Cochrane Library were not searched; this is acknowledged as a limitation in Section 4.6. The full search strings, date limits and record counts are reported in Supplementary Table S2 so that the searches can be reproduced.

### 2.4. Study Selection and Data Extraction

Records were screened by title and abstract, then in full text against the eligibility criteria by two reviewers, with disagreements resolved by consensus with a third author. For each included study, the following were extracted: design, sample size, melanoma subtype, lymphatic and blood endothelial markers and clones, assessed compartments, quantification method (hot-spot, Chalkley or systematic counting; manual or digital), cut-off, effect estimate (odds ratio, hazard ratio, sensitivity and specificity, correlation or difference in means) and adjusted covariates. Multiple reports of the same or overlapping cohort were linked and treated as a single study unit, with each outcome extracted once from the most complete report. The methodological characteristics that drive heterogeneity between studies were tabulated for every study unit (Supplementary Table S3).

### 2.5. Risk-of-Bias Assessment

Risk of bias was assessed for each cutaneous study unit with the Quality In Prognosis Studies (QUIPS) tool across its six domains: study participation, study attrition, prognostic factor measurement, outcome measurement, study confounding, and statistical analysis and reporting [31]. Each domain was rated as low, moderate or high risk by two reviewers, and an overall judgement was derived as low when all domains were rated low or, at most, one was moderate; high when any domain was rated high; and moderate otherwise. The ratings are reported in Section 3.3 and were used to weight the narrative synthesis and the evidence map.

### 2.6. Data Synthesis and Evidence Grading

The primary studies report incompatible effect measures: differences in mean vessel counts between metastasizing and non-metastasizing tumours, odds ratios for nodal metastasis, hazard ratios for survival with differing exposures and cut-offs, and sensitivity and specificity for SLN status. A single harmonised pooled estimate across all studies was therefore not appropriate, a difficulty that the reference meta-analysis also encountered [24,29]. We anchored the quantitative synthesis on the pooled findings of that meta-analysis, extended it narratively with the cohorts published since, and summarised the direction and strength of each association in an evidence map that crosses biomarker and compartment with outcome. Between-study heterogeneity was characterised descriptively from the extracted methodological variables because statistical heterogeneity cannot be quantified without pooling.

The evidence map was graded with explicit, prespecified criteria. An association was graded strong (++) when it was significant in the reference meta-analysis and in at least two subsequent independent cohorts with multivariable adjustment and no cohort reported the opposite direction. It was graded moderate (+) when it was significant in the reference meta-analysis or in at least two independent cohorts, with, at most, one cohort reporting a null or opposite result. It was graded weak or inconsistent (±) when it rested on single cohorts only or when cohorts reported opposite directions and not or insufficient (–) when no significant association was found or fewer than two cohorts were available. Ocular studies and the authors’ own data did not contribute to grading. Two authors graded each cell independently and resolved disagreements by discussion.

## 3. Results

### 3.1. Study Selection

**Figure 1:**
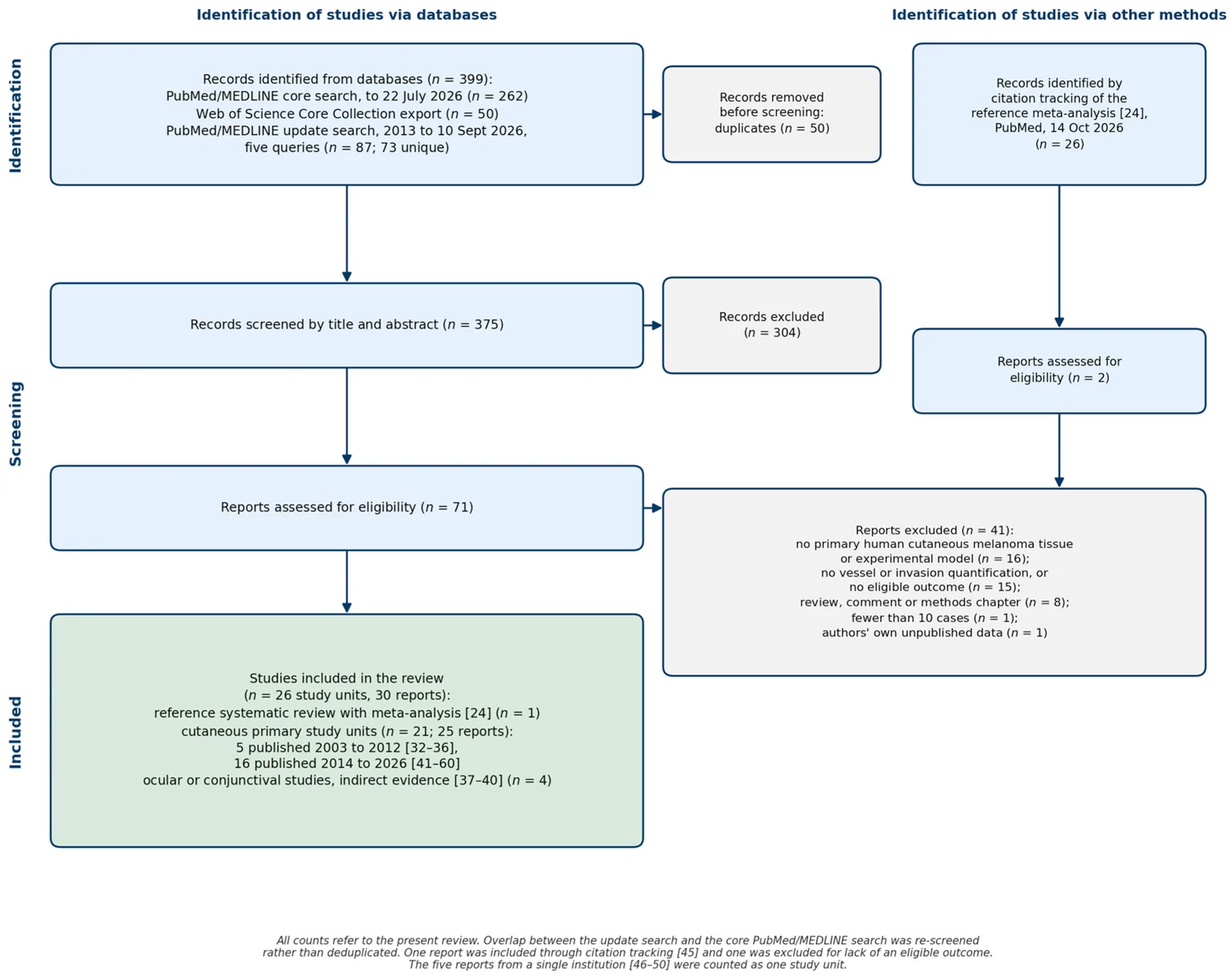
PRISMA 2020 flow diagram of study identification through databases (PubMed/MEDLINE core and update searches, Web of Science export) and through citation tracking of the reference meta-analysis [24]. All counts refer to the present review; one report was included through citation tracking [45], and the five reports from a single institution [46,47,48,49,50] were counted as one study unit. LI, lymphatic invasion; LVD, lymphatic vessel density; MVD, microvascular density.

The study selection process is shown in Figure 1. The core PubMed/MEDLINE search, the Web of Science export and the five update queries identified 399 records, and citation tracking of the reference meta-analysis identified a further 26. After removal of 50 duplicates, 375 records were screened, and 71 reports were assessed in full text. Forty-one reports were excluded, most often because they contained no primary human cutaneous melanoma tissue data or no quantification of vessels or invasion; the authors’ unpublished D2-40/Ki-67 data were excluded under the conflict-of-interest rule in Section 2.2. Thirty reports were included: the reference meta-analysis [24], five cutaneous primary studies from the period it covered [32,33,34,35,36], four ocular or conjunctival studies retained as indirect evidence [37,38,39,40], and twenty reports published between 2014 and 2026 [41,42,43,44,45,46,47,48,49,50,51,52,53,54,55,56,57,58,59,60]. Five of the latter reports from one institution [46,47,48,49,50] describe overlapping specimens and were treated as a single study unit, giving 21 cutaneous study units and 26 study units in total.

### 3.2. Characteristics of the Included Studies

The cutaneous study units are summarised in Table 3 and the ocular studies in Table 4. The cutaneous cohorts ranged from 12 to 321 patients and were, with two exceptions, single-centre and retrospective. The lymphatic endothelium was identified with D2-40 or another anti-podoplanin antibody in 15 study units and with LYVE-1 in six, three of which used both; blood vessels were stained with CD31, CD34, VEGFR-2 or endocan. Quantification was performed by manual hot-spot counting in nine units, by Chalkley counting in two, by computer-assisted morphometry or digital image analysis in six and by multiplex immunofluorescence or multiplex immunohistochemistry in two, with the counting method not stated in two. Peritumoral and intratumoral compartments were defined by the authors’ own conventions, most often as the tumour–stroma interface within a distance of 0.5 to 1 mm, and cut-offs were either study-specific medians, receiver operating characteristic optima or continuous. Effect measures were accordingly varied: odds ratios for nodal metastasis in six units, hazard ratios for survival in six, sensitivity and specificity in two, and differences in means or correlation coefficients in the remainder (Supplementary Table S3). This methodological heterogeneity, rather than any single discordant result, is the main obstacle to pooling.

### 3.3. Risk of Bias in the Included Studies

The QUIPS ratings are shown in Table 5. Five study units were judged at low overall risk of bias [33,35,43,44,60]: they enrolled consecutive or population-based series of more than 150 patients, measured the prognostic factor with a defined protocol; followed patients for several years; and adjusted for thickness, ulceration and other established prognostic variables. Ten units were at moderate risk, most often because of small or selected samples, matched or case–control designs, or limited adjustment for confounding. Six units were at high risk [41,45,53,57,58,59], mainly because they enrolled fewer than 50 patients, reported unadjusted associations with clinicopathological variables rather than with outcome, or were explicitly exploratory. Attrition was rarely a problem because most cohorts were retrospective with complete follow-up, whereas confounding was the domain most frequently rated moderate or high. The evidence map therefore rests mainly on the five low-risk cohorts and on the reference meta-analysis.

### 3.4. Blood Microvascular Density

In the reference meta-analysis, blood MVD (nine studies, 419 patients) did not show consistent prognostic value in cutaneous melanoma [24]. A later systematic review and meta-analysis of MVD reported, at most, a weak and inconsistent association with outcome [14], and reviews of melanoma angiogenesis emphasise that the prognostic signal is marker-dependent and complicated by vasculogenic mimicry and vascular co-option [15,16]. The newer cohorts agree. In 82 melanomas and 25 naevi, MVD rose from naevi to melanoma and correlated with the vertical growth phase, with higher values at the periphery than in the centre, but no survival analysis was performed [45]. In 102 primary melanomas, MVD assessed with VEGFR-2 and endocan correlated with LVD and with thickness and ulceration but not with lymphovascular invasion [42]. In 156 scalp and neck melanomas, blood vessel density exceeded lymphatic density in both compartments, yet only tumour thickness predicted survival [43], and in 196 patients quantified by the Chalkley method, the peritumoral D2-40 score, not the CD34 parameters, emerged as the strongest predictor in multivariable models [44]. Blood endothelial proliferation, measured as CD31/Ki-67 co-expression, was not prognostic in a cohort of 264 patients in which lymphatic proliferation was [60]. In the authors’ published series of 92 melanomas, blood microvessels were most abundant peritumorally, and their density correlated with mast cell density, particularly in the early T categories, consistent with a role for angiogenesis in early growth rather than in dissemination [30].

### 3.5. Lymphatic Microvascular Density by Compartment

The reference meta-analysis found that peritumoral LVD (seven lymphangiogenesis studies, 474 patients) carried prognostic value, whereas intratumoral LVD did not [24]. The earlier primary studies support a peripheral predominance of prognostically relevant lymphatics. Dadras et al. associated tumour lymphangiogenesis and a relative lymphatic vessel area above 1.5% with lymph node metastasis and poorer disease-free and overall survival [32], and lymphatic hot-spot density predicted sentinel node status with 83% sensitivity and 89% specificity, outperforming thickness [34]. Shayan et al. showed that, in metastasizing tumours, peripheral LVD greatly exceeded central LVD (5.73 ± 0.67 vs. 1.72 ± 0.42 vessels per unit area, *p* < 0.001) and that peripheral LVD was higher in metastasizing than in matched non-metastasizing tumours (5.73 ± 0.67 vs. 4.21 ± 0.37, *p* < 0.01) so that the peripheral-to-central ratio marked metastatic propensity [36]. Straume et al., by contrast, reported the opposite direction for absolute intratumoral density in nodular melanoma: decreased intratumoral LVD accompanied thicker, more proliferative tumours, and higher peritumoral LVD was associated with better survival (HR 2.1, *p* = 0.009) [33].

Studies published after the meta-analysis strengthen the peritumoral signal and partly rehabilitate the intratumoral one. In the largest series, 196 patients quantified according to the consensus recommendations for vessel counting, the peritumoral D2-40 Chalkley score was the strongest independent predictor of sentinel node, non-sentinel node and distant metastasis and of disease-free and overall survival [44]. In 100 melanomas from a single centre, computer-assisted morphometry of D2-40 sections showed that both intratumoral (OR of 1.09 per unit) and peritumoral LVD (OR of 1.05 per unit) independently predicted lymph node metastasis, together with VEGF-C expression in tumour cells (OR of 20.3) [48]. In the survival analysis of the same cohort, peritumoral LVD independently predicted disease-free (HR of 2.14, 95% CI 1.10 to 4.19) and melanoma-specific survival (HR of 2.74, 95% CI 1.31 to 5.73), and intratumoral LVD was the strongest predictor of melanoma-specific survival (HR of 3.45, 95% CI 1.47 to 8.11) [50]. The association held only for tumours thicker than 2 mm when metastasizing and non-metastasizing melanomas were matched by thickness [46], and VEGF-C-positive tumours had higher LVD in both compartments [47]. Smaller series point the same way. LYVE-1 LVD in both compartments was higher in node-positive and stage IIIC to IV tumours and correlated with angiopoietin-2 expression [58]; peritumoral lymphatic volume density rose with stage in 40 patients [53]; and lymphatic density was higher peritumorally than intratumorally in facial melanomas, with the highest vascular density in tumours with a dense peritumoral infiltrate [41]. Not every cohort found a prognostic effect. In 102 melanomas, D2-40 LVD tracked thickness and mitoses but not lymphovascular invasion [42]; in scalp and neck melanomas, LVD in neither compartment predicted survival [43]; and a multiplex study of 28 treatment-naive tumours found that the localisation of functional lymphatic subtypes, not overall vessel density, was associated with regional progression [59].

Why do the directions differ? Four methodological differences account for most of the discordance between the Dadras and Straume series and, by extension, across the field. The marker differs: LYVE-1 also labels a subset of macrophages and is down-regulated in some tumour-associated lymphatics, whereas podoplanin labels all lymphatic endothelium but also some stromal cells so that the two antibodies count partly different vessel populations [22,23]. The tumour subtype differs: Straume et al. studied nodular melanomas, in which the expanding tumour mass compresses and effaces intratumoral lymphatics so that a low intratumoral count may indicate a thick, rapidly growing tumour rather than a quiescent one, whereas the matched cohorts of Dadras et al. and the Banja Luka series were dominated by superficial spreading melanomas in which intratumoral lymphatics persist [32,33,50]. The counting method differs: hot-spot counts capture the densest focus, Chalkley counts estimate relative vessel area, and morphometry averages over whole compartments, and these metrics are not interchangeable. Finally, the association is thickness-dependent: in the Banja Luka cohort, the difference between metastasizing and non-metastasizing tumours was confined to tumours thicker than 2 mm [46]. The peritumoral compartment is less affected by these sources of variation because it is not compressed by the tumour mass, is counted in a comparable stromal background in every study and is the compartment through which tumour cells actually leave the lesion, which is the most plausible explanation for its more consistent prognostic signal.

### 3.6. Lymphatic Invasion

Lymphatic invasion was the most consistently prognostic parameter in the reference meta-analysis (ten studies, 802 patients) [24]. Detection depends heavily on method, as recent reviews of the biology of lymphatic invasion emphasise [61]. Yun et al., using D2-40/S-100 in 321 vertical growth-phase melanomas with long follow-up, found that LI and high lymphatic density in areas of complete radial growth-phase regression were associated with shorter time to first metastasis (hazard ratios up to 2.9 and 2.8, respectively) and with melanoma-specific death [35]. Later series confirmed that dual immunohistochemistry for lymphatic endothelium and melanocytes raises the detection rate several-fold over haematoxylin and eosin: from 2% to 26% with S-100/D2-40 in 125 melanomas [54], from 22% with D2-40 alone to 38% with D2-40/MITF in 120 melanomas [51], from 4.9% to 46.5% in 102 melanomas [42] and from 16.6% to 40.4% for lymphovascular invasion overall in scalp and neck tumours [43]. Immunohistochemically detected LI was associated with sentinel node metastasis in each of these cohorts [51,54,56]: in the Boston series, 33% of patients with LI had a positive sentinel node compared with 11% of those without (*p* = 0.006), and progression-free survival was shorter [54]; in 100 melanomas of 1 to 4 mm that were negative for invasion on routine sections, LI detected by dual staining was an independent predictor of sentinel node status and of overall survival [56]. The choice of melanocytic marker matters. D2-40/MART-1 discriminated tumours with lymphatic spread better than D2-40/SOX-10 or D2-40/S-100 (AUC 0.71 vs. 0.58 and 0.63) [57], and combining CD31/S-100 with D2-40/S-100 captured the largest number of positive cases [56]. Angiotropism, the extravascular migration of melanoma cells along the outer surface of vessels, co-occurs with LI and with distant metastasis and may represent a parallel route of spread [55]. One cohort dissented: D2-40/S-100-detected LI alone did not predict nodal status (AUC 0.56), whereas a composite index combining thickness, peritumoral LVD and LI did (AUC 0.86) [49]. Lymphatic invasion is also a recognised predictor of non-sentinel node metastasis [62].

### 3.7. Proliferating (Ki-67-Positive) Lymphatic Vessels

Resolving the actively proliferating lymphatic fraction with Ki-67 provides information beyond a static vessel count. The concept was first developed in melanocytic tumours of the ocular adnexa, where proliferating (Ki-67-positive) lymphatic vessels increased with progression from premalignant lesions to invasive melanoma and, when abundant intratumorally, were associated with lower recurrence-free, metastasis-free and melanoma-specific survival (Table 4) [37,38,39,40]. These ocular data are indirect: the conjunctiva and uvea differ from skin in their resident lymphatics and immune environment, and they were not used for grading.

Cutaneous data were scarce at the time of the reference meta-analysis but are no longer. In 196 patients, CD34/Ki-67 and D2-40/Ki-67 double staining quantified endothelial proliferation fractions separately in the peritumoral and intratumoral compartments, and the peritumoral lymphatic Chalkley score dominated the multivariable models [44]; in matched primary tumours and sentinel node metastases from 44 patients, the primary tumour showed a higher rate of lymphatic proliferation than its nodal metastasis, and the rate of peritumoral lymphatic proliferation was inversely related to non-sentinel node involvement [52]. The strongest evidence comes from a cohort of 264 patients with a single invasive primary melanoma and a median follow-up of 7.1 years, in which Ki-67 within D2-40-positive endothelium, detected by multiplex immunofluorescence, predicted melanoma-specific mortality (aHR of 2.03, 95% CI 1.33 to 3.10) and recurrence (aHR of 1.70) after adjustment for age, sex, Breslow depth, ulceration and anatomical site, whereas Ki-67 within CD31-positive blood endothelium did not [60]. Tumour cell proliferation and peritumoral vessel density also rose in parallel with stage in a smaller series of 40 patients [53]. Taken together, two independent cutaneous cohorts with survival end-points now support the proliferating lymphatic fraction as a prognostic marker, although neither reported distant metastasis separately.

### 3.8. Evidence Map

**Figure 2:**
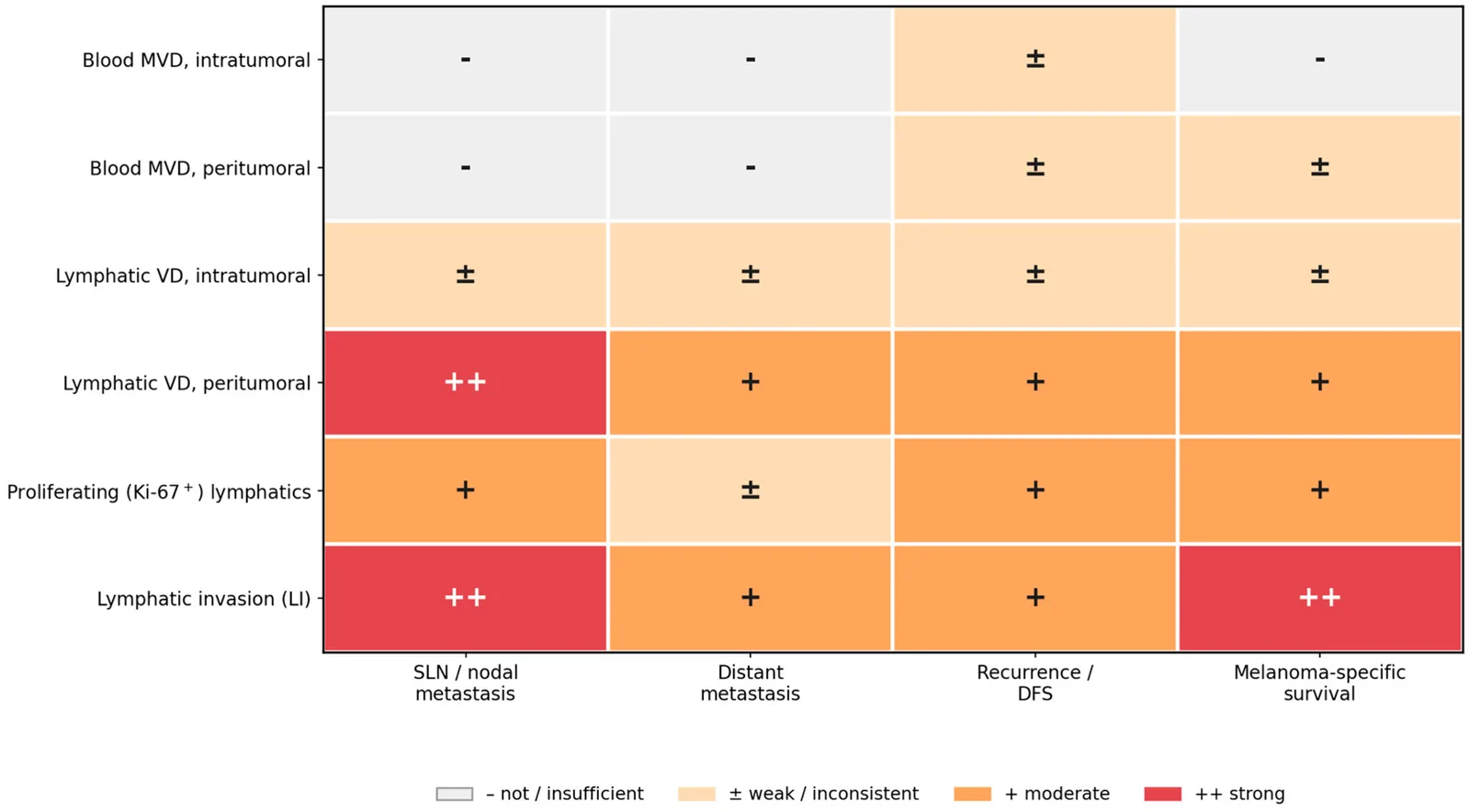
Evidence map of prognostic associations by biomarker and compartment (rows) and outcome (columns), graded with the criteria of Section 2.6: - not or insufficient; ± weak or inconsistent; + moderate; ++ strong. Ocular studies and the authors’ own data did not contribute. DFS, disease-free survival; LI, lymphatic invasion; MVD, microvascular density; SLN, sentinel lymph node; VD, vessel density.

The direction and strength of the prognostic associations across biomarker, compartment and outcome, graded by the criteria of Section 2.6, are summarised in Figure 2. Peritumoral LVD and LI reach the strong grade for nodal metastasis, and LI is also graded as strong for melanoma-specific survival because each is supported by the pooled estimate and by at least two adjusted cohorts in the same direction. Peritumoral LVD is graded moderate for distant metastasis and for survival because of the opposite direction reported in the nodular series [33]. Intratumoral LVD is inconsistent throughout: it was prognostic in the single-centre morphometric cohort [50] but not in the pooled analysis [24] or in two other recent series [42,43], and the nodular series pointed the other way [33]. Blood MVD is graded not or insufficient for metastasis and survival. Proliferating lymphatics are graded moderate for nodal metastasis, recurrence and melanoma-specific survival on the basis of two independent cutaneous cohorts [44,60] and weak for distant metastasis, for which only one cutaneous cohort reported data.

## 4. Discussion

### 4.1. Principal Findings

Synthesised across the reference meta-analysis and the cohorts published since, the evidence indicates that the prognostically informative lymphatic signal in cutaneous melanoma resides at the tumour periphery, in lymphatic invasion and in the proliferating lymphatic fraction rather than in blood microvessel density or in the absolute intratumoral lymphatic count [24,44,50,54,60]. The effect sizes are clinically meaningful: adjusted hazard ratios of roughly 2 to 3.5 for melanoma-specific survival are of the same order as those attached to ulceration or to a one-category increase in T stage [4]. This spatial pattern is biologically coherent. Peritumoral lymphatics at the invasive front provide the functional route for tumour cell entry into the lymphatic system, and their expansion, together with lymphatic invasion, reflects a metastasis-permissive microenvironment [17,18]. Lymph fluid itself favours the survival of disseminating melanoma cells by protecting them from ferroptosis, which helps to explain why the lymphatic route is preferred over the blood-borne one [63]. Tumour-derived extracellular vesicles, including NGFR-positive vesicles that induce lymphangiogenesis, and secreted factors then remodel the draining node into a pre-metastatic niche before tumour cells arrive [64,65,66].

### 4.2. Reconciling Discordant Results

The compartmental picture is less uniform than the headline finding suggests. Whereas the pooled evidence finds intratumoral LVD non-prognostic [24], individual studies report divergent directions depending on marker, cut-off, quantification method and tumour subtype, as analysed in Section 3.5: intratumoral lymphangiogenesis was adverse in the matched cohorts of Dadras et al. [32] and in the morphometric single-centre series [50], but decreased absolute intratumoral density accompanied thicker tumours in the nodular series of Straume et al. [33], and overall density carried no signal once vessel subtypes were resolved by multiplex imaging [59]. Resolving the proliferating fraction with Ki-67 may reconcile part of this heterogeneity because it is the actively growing lymphatic vasculature, rather than the static count, that tracks with aggressiveness in ocular melanoma [39,40] and with survival in the two cutaneous cohorts [44,60]. Recent experimental work supports this reading. Interferon-gamma signalling restrains an immature, proliferative, tip-cell-like lymphatic state that promotes lymph-node metastasis without supporting dendritic cell migration or the response to checkpoint blockade, and intratumoral lymphatic density is inversely related to cytotoxic immunity [67]. A compressed, non-proliferating intratumoral network and an expanding, proliferating peritumoral network are therefore two faces of the same aggressive tumour, and a single global density conflates them. These observations motivate the reporting of compartment-specific and proliferation-resolved lymphatic metrics.

### 4.3. Lymphatic Vessels, Immunity and Mechanism

Tumour lymphangiogenesis is driven mainly by VEGF-C and VEGF-D signalling through VEGFR-3 and is amplified by an inflammatory infiltrate in which tumour-associated macrophages supply VEGF-C and VEGF-D; lymphatic vessel number correlates with the intensity of peritumoral inflammation [17,18,35,41]. Tumour cell VEGF-C and VEGFR-3 expression have, themselves, predicted nodal metastasis and survival in cutaneous cohorts [48,68,69], and the VEGF/VEGFR axis remains the main target under study in terms of interfering with melanoma lymphangiogenesis [70]. However, the relationship between tumour lymphatics and immunity is two-sided. Lymphatic vessels are required for antigen and dendritic cell transport to the draining node and for the establishment of tumour-associated inflammation [21,71]; in human primary melanomas, LVD correlates with CD8-positive T-cell infiltration but also with immunosuppressive enzymes IDO and iNOS [72]. In mouse melanoma, VEGF-C-driven lymphangiogenesis increased naive T-cell infiltration and potentiated immunotherapy, and serum VEGF-C predicted the response to checkpoint blockade in patients [73]. The lymphatic endothelium also expresses PD-L1 and MHC class II and can restrain tumour-specific CD8-positive T cells [74,75], yet oxysterols derived from lymphatic endothelial cells promote pro-inflammatory myeloid cells, and their signature is associated with better survival and immunotherapy response in human melanoma [76]. A high lymphatic vessel density therefore signals both metastatic risk and an immunologically engaged tumour, and the prognostic weight of LVD is likely to shift under adjuvant immunotherapy [27].

### 4.4. Clinical Implications: Sentinel Node Biopsy, Staging and Follow-Up

How would these markers change management? The first decision they could inform is whether to offer sentinel lymph node biopsy (SLNB). Current guidelines recommend SLNB from a thickness of 0.8 mm or in thinner ulcerated tumours and discuss it case by case in T1a tumours with adverse features [77,78]. In thin melanoma, the pooled positivity rate is only 5.1%, rising to about 7% with a thickness of 0.8 mm or more, mitoses or ulceration [79], so most patients undergo a procedure whose result is negative, while SLNB, itself, has a pooled sensitivity of 0.85 and a negative predictive value above 0.93 [80]. Peritumoral LVD and immunohistochemically detected LI are measured on the diagnostic excision, before any staging procedure, and in the cohorts reviewed here, they identified groups with a threefold higher nodal positivity [51,54,56] or predicted sentinel node status more accurately than thickness alone [34,44,49]. Their most plausible use is therefore as adjuncts in the borderline T1b to T2a range, where a high peritumoral LVD or LI would support offering SLNB and their absence would support omitting it, in the same way that mitotic rate and ulceration are used today; among the tissue biomarkers proposed for predicting nodal status, they are the only ones that can be read on routine immunohistochemistry without molecular testing [81]. A composite index of thickness, peritumoral LVD and LI discriminated nodal status with an AUC of 0.86 in one cohort [49], but none of these markers has been validated prospectively, and the external validation of existing SLN prediction tools shows that discrimination does not guarantee calibration in a new population [62].

The second decision is what to do after a positive sentinel node. Completion lymphadenectomy no longer improves melanoma-specific survival [82,83], and SLNB, itself, improves survival only in the subgroup with intermediate-thickness tumours and nodal metastasis [84], so the information that LI and peritumoral LVD provide about non-sentinel node involvement [44,62] now matters for surveillance and adjuvant decisions rather than for surgery. Adjuvant anti-PD-1 therapy is approved for resected stage III and, since the KEYNOTE-716 and CheckMate 76K trials, for stage IIB and IIC melanoma, where it reduces recurrence by more than a third [6,7,8,85,86]. A node-negative stage IB or IIA patient whose primary shows LI and a high proliferating peritumoral LVD has, on the basis of the evidence reviewed here, a risk of recurrence and melanoma-specific death that approaches that of stage IIB and would be a candidate for trials that extend adjuvant therapy or intensified imaging to lower stages; conversely, a stage IIB patient without any lymphatic marker might be a candidate for de-escalation studies. The third decision is the intensity of follow-up. European guidelines propose a stage-based scheme but acknowledge that there is no evidence to define its frequency [78]; markers that identify the minority of early-stage patients who relapse would allow lymph node ultrasound and imaging to be concentrated on them.

### 4.5. Prognostic Markers and the Outcome of Therapy

The vascular markers reviewed here have also been examined as predictors of treatment effect, with sobering results for anti-angiogenic therapy. Adjuvant bevacizumab did not improve overall survival in 1343 patients with stage IIB to III melanoma in the AVAST-M trial, despite a modest gain in disease-free interval, and no vascular biomarker identified a benefiting subgroup [87]; this concords with the absence of a prognostic signal for blood MVD in the present review. Anti-lymphangiogenic agents directed at VEGF-C and VEGFR-3 have not progressed beyond preclinical models, and the finding that VEGF-C signalling potentiates rather than blunts the response to checkpoint blockade [73] argues against blocking it in patients who receive immunotherapy. Combinations of anti-angiogenic agents with checkpoint inhibitors are being tested on the premise that vascular normalisation improves T-cell infiltration, with the most consistent signals so far in mucosal and acral disease [16,88]. For the lymphatic markers, the relevant question is predictive rather than therapeutic: because lymphatic vessels carry antigen and dendritic cells to the draining node and because T-cell infiltration associated with lymphatic vessels and oxysterol signalling are associated with better immunotherapy outcomes [72,76], a tumour with abundant, proliferating peritumoral lymphatics may be both more likely to metastasize and more likely to respond to adjuvant anti-PD-1 therapy. No adjuvant trial has yet stratified patients by LVD, LI or endothelial proliferation, and the tissue collections of KEYNOTE-716, CheckMate 76K and the stage III trials would be the natural place to test whether these markers predict benefit. From a diagnostic standpoint, the D2-40/Ki-67 double stain is inexpensive and routinely feasible, and digital pathology with open-source tools such as QuPath (Queen’s University Belfast, Belfast, UK) could standardise vessel quantification and enable composite spatial biomarkers that integrate lymphatic, blood-vascular and immune features [59,69,89].

### 4.6. Strengths and Limitations

The strengths of this review are its explicit eligibility and grading criteria, the formal risk-of-bias assessment, the separation of ocular from cutaneous evidence, the exclusion of the authors’ own unpublished data from the evidence base, and an update search that added 16 cutaneous study units to the five available to the reference meta-analysis. Its principal limitation is the scope of the search: PubMed/MEDLINE was the only database searched systematically, supplemented by a Web of Science export and by citation tracking, and Embase, Scopus and the Cochrane Library were not searched, so conference reports and journals not indexed in MEDLINE may have been missed. The review was not prospectively registered. The second limitation is methodological heterogeneity: studies differ in marker and clone, compartment definition, hot-spot versus Chalkley versus morphometric counting, cut-offs and the reported effect measure (Supplementary Table S3), which constrained the synthesis to a narrative update of the 2014 pooled analysis rather than a new meta-analysis; for this reason, the title no longer claims a meta-analytic update. Meta-analyses in breast, oesophageal, hepatobiliary and oral cancers have nevertheless shown that LVD can be pooled once cohorts report hazard ratios with comparable definitions [90,91,92,93], and the newer melanoma cohorts that report adjusted hazard ratios for peritumoral LVD and for proliferating lymphatics [44,50,60] make an updated pooled estimate feasible once standardised aggregate or individual patient data can be obtained. Third, several of the newer series are single-centre and retrospective, five reports from one institution describe overlapping specimens and were counted once, and five study units were at high risk of bias. Finally, the evidence map is a structured expert judgement; its criteria are stated so that readers can disagree with individual cells.

## 5. Conclusions

Current evidence supports peritumoral lymphatic vessel density; lymphatic invasion; and, on the strength of two recent cutaneous cohorts, the proliferating (Ki-67-positive) lymphatic fraction as prognostic biomarkers in primary cutaneous melanoma, whereas blood microvascular density and absolute intratumoral lymphatic density are not consistently informative, in agreement with the reference meta-analysis. Measured on the diagnostic excision with inexpensive stains, these markers could refine the selection of patients for sentinel lymph node biopsy in the borderline thickness range, identify node-negative patients at stage IIB-like risk who might benefit from adjuvant therapy or intensified follow-up, and possibly predict the response to adjuvant immunotherapy. Resolving the intratumoral and peritumoral compartments and the proliferating fraction with D2-40/Ki-67 is a feasible refinement that merits prospective, protocol-registered evaluation with standardised, digital-pathology-based quantification and with immune markers measured alongside, given the dual role of tumour lymphatics in metastasis and antitumour immunity. An updated meta-analysis with harmonised effect measures is warranted and becomes feasible as cohorts reporting adjusted hazard ratios accumulate.

## Figures and Tables

**Table 1:** PECO eligibility framework for the systematic review.

Element	Definition
Population	Patients with histopathologically confirmed primary cutaneous melanoma; ocular, conjunctival and mucosal melanoma retained only as indirect evidence
Exposure	Blood MVD, lymphatic vessel density, proliferating (Ki-67-positive) lymphatic vessels or lymphatic invasion, assessed by immunohistochemistry or digital pathology, with intratumoral versus peritumoral localisation where reported
Comparator	High versus low density; metastatic versus non-metastatic tumours; peritumoral versus intratumoral compartment
Outcomes	Sentinel lymph node, regional or distant metastasis; recurrence or disease-free survival; melanoma-specific or overall survival; association with established prognostic variables (Breslow thickness, ulceration, stage)

**Table 2:** Core PubMed/MEDLINE search concept blocks (combined with AND).

Concept Block	Representative Terms
Disease	“Melanoma”[Mesh] OR melanoma * [tiab] OR “cutaneous melanoma” [tiab]
Vascular/lymphatic	angiogenesis OR microvessel * OR “vascular density” OR CD31 OR CD34 OR CD105 OR lymphangiogenesis OR “lymphatic vessel *” OR podoplanin OR “D2-40” OR LYVE-1 OR VEGFR-3 OR VEGF-C OR VEGF-D
Spatial (omitted in the sensitised version)	intratumoral OR peritumoral OR “invasive front” OR “tumour edge” OR compartment * OR spatial

* Truncation (wildcard) symbol in PubMed search syntax.

**Table 3:** Cutaneous primary study units and the reference meta-analysis included in the synthesis, in chronological order.

Study	Year	Design/n	Marker (s) and Principal Finding
Pastushenko et al. [24]	2014	Meta-analysis (9 MVD, 7 LVD and 10 LI studies; 419/474/802 patients)	IHC MVD/LVD/LI: peritumoral LVD and LI associated with metastasis; MVD and intratumoral LVD not prognostic
Dadras et al. [32]	2003	18 metastatic vs. 19 non-metastatic, matched	LYVE-1/CD31: intratumoral lymphangiogenesis higher in metastatic tumours; relative lymphatic area >1.5% associated with poorer DFS and OS
Straume et al. [33]	2003	Nodular melanoma cohort	LYVE-1/podoplanin: median LVD of 6.3 (intratumoral) and 12.5 (peritumoral) per mm^2^; low intratumoral LVD in thick tumours; higher peritumoral LVD associated with better survival (HR of 2.1, *p* = 0.009)
Dadras et al. [34]	2005	45 primary melanomas with SLNB	LYVE-1 hot-spots: LVD predicted SLN metastasis (sensitivity 83%, specificity 89%), outperforming thickness
Yun et al. [35]	2011	321 VGP melanomas, follow-up ≥10 y	D2-40/S-100: high LVD, time to first metastasis HR of 2.8 and melanoma-specific death HR of 2.6; LI HR of 2.9
Shayan et al. [36]	2012	11 metastasizing vs. 11 non-metastasizing, matched	Podoplanin: peripheral LVD of 5.73 ± 0.67 vs. central 1.72 ± 0.42 (metastasizing, *p* < 0.001); peripheral-to-central ratio marks metastatic risk
Toader et al. [41]	2014	12 facial melanomas	D2-40/CD31: LVD higher peritumorally; intratumoral LVD correlated with MVD; highest vessel density with dense peritumoral infiltrate
Aung et al. [42]	2015	102 primary melanomas	D2-40 LVD, VEGFR-2/endocan MVD, D2-40/S-100 LVI: LVD associated with thickness and mitoses; IHC raised LVI detection from 4.9% to 46.5%; MVD and LVD not associated with LVI
Pasquali et al. [43]	2015	156 scalp and neck melanomas, multicentre	IHC LVI of 34.6% and BVI of 13.5% (lymphovascular invasion 40.4% vs. 16.6% by morphology); BVD exceeded LVD in both compartments; only thickness predicted survival
Pastushenko et al. [44]	2015	196 melanomas	CD34/Ki-67 and D2-40/Ki-67 Chalkley counts: peritumoral D2-40 Chalkley score strongest independent predictor of SLN; non-SLN; and distant metastasis, DFS and OS
Zidlik et al. [45]	2015	82 melanomas (pT1 to pT4) and 25 naevi	MVD, CD3-positive TILs and FOXP3-positive cells in centre and periphery: all higher in melanoma than in naevi and correlated with VGP; topographic differences between centre and periphery
Špirić et al. [46,47,48,49,50]	2015 to 2020	100 melanomas, single centre (five overlapping reports; 95 melanomas + 22 naevi and 54 melanomas in subsets)	D2-40 morphometry: intratumoral (OR 1.09) and peritumoral LVD (OR 1.05) and VEGF-C (OR 20.3) predicted nodal metastasis; peritumoral LVD predicted DFS (HR 2.14) and MSS (HR 2.74), intratumoral LVD predicted MSS (HR 3.45); D2-40/S-100 LI alone not predictive (AUC 0.56)
Feldmeyer et al. [51]	2016	120 primary melanomas	D2-40 with or without MITF LVI: 22% vs. 38% detection; LVI associated with thickness, mitoses and SLN metastasis
Pastushenko et al. [52]	2016	44 primaries with matched SLN metastases	CD34/Ki-67 and D2-40/Ki-67: lymphatic proliferation higher in primary than in nodal metastasis; peritumoral lymphatic proliferation inversely related to non-SLN involvement
Bgatova et al. [53]	2016	40 melanomas	Podoplanin/LYVE-1/CD34/Ki-67: peritumoral blood and lymphatic vessel volume density and tumour proliferation rose with stage
Moy et al. [54]	2017	125 melanomas with SLNB	S-100/D2-40 LI: 2% (H&E) vs. 26% detection; SLN positive in 33% with LI vs. 11% without; shorter progression-free survival
Moy et al. [55]	2019	179 primary melanomas	H&E, CD31 and S-100/D2-40: angiotropism in 17% to 37% depending on method; associated with LI, mitotic rate, progression and distant metastasis (univariable)
Bayram et al. [56]	2021	100 melanomas 1 to 4 mm, LVI-negative on H&E	CD31/S-100 (40%), D2-40/S-100 (35%), and CD34/S-100 (17%): LVI independent predictor of SLN status and OS
Straker et al. [57]	2022	22 with vs. 11 without lymphatic spread	D2-40 paired with MART-1, SOX-10 or S-100: D2-40/MART-1 most accurate for LI (AUC 0.71)
Zheng et al. [58]	2023	42 melanomas	LYVE-1 LVD (peri- and intratumoral): higher with nodal metastasis and stage IIIC to IV; correlated with angiopoietin-2 and Breslow thickness
Femel et al. [59]	2024	28 treatment-naive primaries	Quantitative multiplex IHC: five lymphovascular subtypes; localisation of subtypes, not overall density, associated with lymphoid aggregates, regional progression and T-cell infiltration
Tan et al. [60]	2024	264 single primary melanomas with SLNB; median follow-up of 7.1 y	Multiplex IF D2-40/Ki-67 and CD31/Ki-67: lymphatic Ki-67 predicted MSS (aHR 2.03) and recurrence (aHR 1.70); blood endothelial Ki-67 not prognostic

aHR, adjusted hazard ratio; AUC, area under the receiver operating characteristic curve; BVD, blood vessel density; BVI, blood vessel invasion; DFS, disease-free survival; H&E, haematoxylin and eosin; HR, hazard ratio; IF, immunofluorescence; IHC, immunohistochemistry; LI, lymphatic invasion; LVD, lymphatic vessel density; LVI, lymphovascular invasion; MSS, melanoma-specific survival; MVD, microvascular density; OR, odds ratio; OS, overall survival; SLN, sentinel lymph node; SLNB, sentinel lymph node biopsy; TIL, tumour-infiltrating lymphocyte; VGP, vertical growth phase.

**Table 4:** Ocular and conjunctival melanoma studies retained as indirect evidence on proliferating lymphatic vessels (not used for grading).

Study	Year	Design/n	Marker (s) and Principal Finding
Zimmermann et al. [37]	2009	20 conjunctival melanomas	LYVE-1/podoplanin/Ki-67: intra- and peritumoral proliferating lymphatics documented
Heindl et al. [38]	2010	20 ciliary body melanomas	LYVE-1/podoplanin: intraocular lymphatics associated with higher mortality (HR 8.09, *p* = 0.04)
Heindl et al. [39]	2011	109 conjunctival melanomas	Podoplanin/Ki-67: high intratumoral lymphatic density associated with worse recurrence, spread, distant metastasis and survival
Heindl et al. [40]	2011	20 conjunctival melanomas and precursors	Proliferating (Ki-67-positive) lymphatics increased with progression; high intratumoral density associated with worse survival

**Table 5:** Risk of bias of the cutaneous study units assessed with the Quality In Prognosis Studies (QUIPS) tool.

Study Unit	1	2	3	4	5	6	Overall
Dadras 2003 [32]	M	L	L	L	M	M	Moderate
Straume 2003 [33]	L	M	M	L	L	L	Low
Dadras 2005 [34]	M	L	M	L	M	M	Moderate
Yun 2011 [35]	L	L	L	L	L	L	Low
Shayan 2012 [36]	M	L	L	L	M	M	Moderate
Toader 2014 [41]	H	L	M	H	H	H	High
Aung 2015 [42]	M	L	L	M	M	M	Moderate
Pasquali 2015 [43]	L	M	L	L	L	L	Low
Pastushenko 2015 [44]	L	L	L	L	L	L	Low
Zidlik 2015 [45]	M	L	M	M	H	M	High
Špirić 2015 to 2020 [46,47,48,49,50]	M	M	L	L	M	M	Moderate
Feldmeyer 2016 [51]	M	M	L	L	M	M	Moderate
Pastushenko 2016 [52]	M	L	L	L	M	M	Moderate
Bgatova 2016 [53]	H	L	M	M	H	H	High
Moy 2017 [54]	M	L	L	L	M	M	Moderate
Moy 2019 [55]	M	L	L	L	M	L	Moderate
Bayram 2021 [56]	M	M	L	L	L	M	Moderate
Straker 2022 [57]	H	L	L	M	H	M	High
Zheng 2023 [58]	H	L	M	M	H	M	High
Femel 2024 [59]	H	L	L	M	M	M	High
Tan 2024 [60]	L	M	L	L	L	L	Low

H, high risk of bias; L, low risk of bias; M, moderate risk of bias. Domains: 1, study participation; 2, study attrition; 3, prognostic factor measurement; 4, outcome measurement; 5, study confounding; 6, statistical analysis and reporting.

## Data Availability

All analysed data are contained within the cited published articles and in the Supplementary Materials (search strings, PRISMA checklist and extraction table).

## Supplementary Materials

The following supporting information can be downloaded at: https://www.mdpi.com/article/10.3390/cancers18203412/s1, Table S1: PRISMA 2020 checklist; Table S2: full search strings, date limits and record counts; Table S3: methodological characteristics of the included cutaneous study units.

## Abbreviations

The following abbreviations are used in this manuscript:

LVD	Lymphatic vessel density
MVD	Microvascular density
LI	Lymphatic invasion
VEGF(R)	Vascular endothelial growth factor (receptor)
SLN	Sentinel lymph node
DFS	Disease-free survival
QUIPS	Quality In Prognosis Studies
PRISMA	Preferred Reporting Items for Systematic Reviews and Meta-Analyses
LVI	Lymphovascular invasion
MSS	Melanoma-specific survival
OS	Overall survival
SLNB	Sentinel lymph node biopsy
AJCC	American Joint Committee on Cancer
