# Supplementary material for: Lymphatic Microvascular Density, Lymphatic Invasion and Endothelial Cell Proliferation as Prognostic Biomarkers in Cutaneous Melanoma: A Systematic Review and Evidence Map
Source: Cancers (Basel). 2026 Oct 21;18(20):3412. doi: 10.3390/cancers18203412 (PMC13702970; doi:10.3390/cancers18203412)
Supplement: Supplementary file 1 [file cancers-18-03412-s001.zip › cancers-4618689-supplementary.pdf]

Supplementary Materials

Lymphatic Microvascular Density, Lymphatic Invasion and Endothelial Cell Proliferation as Prognostic Biomarkers in Cutaneous Melanoma: A Systematic Review and Evidence Map (Cancers, manuscript cancers-4618689). Reference numbers in square brackets refer to the reference list of the main text.

Table S1. PRISMA 2020 checklist

Page MJ et al. BMJ 2021;372:n71 [28]. Locations refer to sections of the manuscript.

Section and topic	Item	Checklist item	Location where item is reported
Title	1	Identify the report as a systematic review.	Title
Abstract	2	See the PRISMA 2020 for Abstracts checklist.	Abstract
Rationale	3	Describe the rationale for the review in the context of existing knowledge.	Section 1, paragraphs 1 to 4
Objectives	4	Provide an explicit statement of the objective(s) or question(s) the review addresses.	Section 1, last paragraph
Eligibility criteria	5	Specify the inclusion and exclusion criteria for the review and how studies were grouped for the syntheses.	Section 2.2, Table 1
Information sources	6	Specify all databases, registers, websites, organisations, reference lists and other sources searched or consulted, and the date when each source was last searched.	Section 2.3, Table S2
Search strategy	7	Present the full search strategies for all databases, registers and websites, including any filters and limits used.	Table 2, Table S2
Selection process	8	Specify the methods used to decide whether a study met the inclusion criteria, including how many reviewers screened each record and each report retrieved.	Section 2.4
Data collection process	9	Specify the methods used to collect data from reports.	Section 2.4
Data items	10a	List and define all outcomes for which data were sought.	Section 2.2, Table 1
Data items	10b	List and define all other variables for which data were sought.	Section 2.4, Table S3
Study risk of bias assessment	11	Specify the methods used to assess risk of bias in the included studies.	Section 2.5
Effect measures	12	Specify for each outcome the effect measure(s) used in the synthesis or presentation of results.	Section 2.6, Table S3
Synthesis methods	13a	Describe the processes used to decide which studies were eligible for each synthesis.	Sections 2.2 and 2.6
Synthesis methods	13b	Describe any methods required to prepare the data for presentation or synthesis.	Section 2.6 (no data conversion; no pooling)
Synthesis methods	13c	Describe any methods used to tabulate or visually display results of individual studies and syntheses.	Section 2.6, Tables 3 to 5, Figure 2
Synthesis methods	13d	Describe any methods used to synthesise results and provide a rationale for the choice(s).	Section 2.6 (narrative synthesis anchored on the 2014 meta-analysis; no new meta-analysis)
Synthesis methods	13e	Describe any methods used to explore possible causes of heterogeneity among study results.	Sections 2.6 and 3.2, Table S3
Synthesis methods	13f	Describe any sensitivity analyses conducted to assess robustness of the synthesized results.	Not applicable (no pooling)
Reporting bias assessment	14	Describe any methods used to assess risk of bias due to missing results in a synthesis.	Not assessed; acknowledged in Section 4.6
Certainty assessment	15	Describe any methods used to assess certainty (or confidence) in the body of evidence for an outcome.	Section 2.6 (evidence map grading criteria)
Study selection	16a	Describe the results of the search and selection process, ideally using a flow diagram.	Section 3.1, Figure 1
Study selection	16b	Cite studies that might appear to meet the inclusion criteria, but which were excluded, and explain why.	Section 3.1, Figure 1 (reasons)
Study characteristics	17	Cite each included study and present its characteristics.	Section 3.2, Tables 3 and 4

Risk of bias in studies	18	Present assessments of risk of bias for each included study.	Section 3.3, Table 5
Results of individual studies	19	For all outcomes, present for each study summary statistics and an effect estimate and its precision.	Sections 3.4 to 3.7, Tables 3 and 4
Results of syntheses	20a	For each synthesis, briefly summarise the characteristics and risk of bias among contributing studies.	Sections 3.2 and 3.3
Results of syntheses	20b	Present results of all statistical syntheses conducted.	Sections 3.4 to 3.8, Figure 2 (narrative synthesis)
Results of syntheses	20c	Present results of all investigations of possible causes of heterogeneity among study results.	Section 3.5, last paragraph; Section 4.2
Results of syntheses	20d	Present results of all sensitivity analyses conducted.	Not applicable
Reporting biases	21	Present assessments of risk of bias due to missing results for each synthesis assessed.	Not assessed; Section 4.6
Certainty of evidence	22	Present assessments of certainty (or confidence) in the body of evidence for each outcome assessed.	Section 3.8, Figure 2
Discussion	23a	Provide a general interpretation of the results in the context of other evidence.	Sections 4.1 to 4.3
Discussion	23b	Discuss any limitations of the evidence included in the review.	Sections 3.3 and 4.6
Discussion	23c	Discuss any limitations of the review processes used.	Section 4.6
Discussion	23d	Discuss implications of the results for practice, policy, and future research.	Sections 4.4, 4.5 and 5
Registration and protocol	24a	Provide registration information for the review, or state that the review was not registered.	Section 2.1 (not registered)
Registration and protocol	24b	Indicate where the review protocol can be accessed, or state that a protocol was not prepared.	Section 2.1 (no protocol prepared)
Registration and protocol	24c	Describe and explain any amendments to information provided at registration or in the protocol.	Not applicable
Support	25	Describe sources of financial or non-financial support for the review, and the role of the funders or sponsors in the review.	Funding statement
Competing interests	26	Declare any competing interests of review authors.	Conflicts of Interest statement
Availability of data, code and other materials	27	Report which of the following are publicly available and where they can be found.	Data Availability Statement; Tables S2 and S3

Table S2. Full search strings, date limits and record counts

All PubMed/MEDLINE searches used the standard PubMed interface; date limits refer to the publication date field. The update queries were run on 10 September 2026; citation tracking was performed with the PubMed "Cited by" function on 14 October 2026. The asterisk is the PubMed truncation (wildcard) operator.

Source and date	Query	Records	Full text	Included
PubMed/MEDLINE core search, inception to 22 July 2026	("Melanoma"[Mesh] OR melanoma*[tiab] OR "cutaneous melanoma"[tiab]) AND (angiogenesis OR microvessel* OR "vascular density" OR CD31 OR CD34 OR CD105 OR lymphangiogenesis OR "lymphatic vessel*" OR podoplanin OR "D2-40" OR LYVE-1 OR VEGFR-3 OR VEGF-C OR VEGF-D) AND (intratumoral OR peritumoral OR "invasive front" OR "tumour edge" OR "tumor edge" OR compartment* OR spatial); sensitised version run without the third block	262	35	10: reference meta-analysis [24], 5 cutaneous studies [32–36], 4 ocular studies [37–40]
Web of Science Core Collection export, 22 July 2026	Same concept blocks (topic search), most recent 50 records exported	50	0	0
PubMed update query 1 (2013/01/01 to 2026/09/10)	(melanoma[Title]) AND (lymphatic vessel density[Title/Abstract] OR lymphatic microvessel density[Title/Abstract] OR lymphangiogenesis[Title]) AND (D2-40 OR podoplanin OR LYVE-1) AND (prognos* OR metasta* OR sentinel)	12		
PubMed update query 2 (2014/01/01 to 2026/09/10)	(cutaneous melanoma[Title/Abstract]) AND (lymphatic invasion[Title/Abstract] OR lymphovascular invasion[Title/Abstract]) AND (prognos* OR sentinel OR survival) AND (immunohistochem* OR D2-40 OR podoplanin)	17		
PubMed update query 3 (2012/01/01 to 2026/09/10)	(melanoma[Title/Abstract]) AND (Ki-67 OR Ki67 OR proliferating) AND (lymphatic vessel* OR lymphatic endothel*) AND (podoplanin OR D2-40 OR LYVE-1) NOT (conjunctiv* OR ocular OR uveal)	8		
PubMed update query 4 (2014/01/01 to 2026/09/10)	(melanoma[Title]) AND (VEGF-C[Title/Abstract] OR VEGFR-3[Title/Abstract] OR VEGF-D[Title/Abstract]) AND (immunohistochem* OR expression) AND (prognos* OR metasta* OR sentinel OR survival) AND (patients OR cohort OR cases)	12		
PubMed update query 5 (2013/01/01 to 2026/09/10)	(melanoma[Title]) AND (peritumoral OR intratumoral OR invasive front OR tumour edge OR spatial) AND (lymphatic vessel* OR lymphatics OR podoplanin OR D2-40) AND (immunohistochem* OR staining) NOT Review[Publication Type]	38		
Update queries 1 to 5 combined	Union of the five queries after removal of 14 within-search duplicates; overlap with the core search was re-screened rather than deduplicated	73 unique	34	19 reports, 15 study units [41–44,46–60]
Citation tracking, PubMed "Cited by", 14 October 2026	Records citing the reference meta-analysis, Pastushenko et al. 2014 [24] (PMID 24134623); 2 already retrieved by the update search	26	2	1: Zidlik et al. 2015 [45]; Bordry et al. 2018 [72] excluded (no eligible outcome)
Reference lists of included reports	Backward citation screening of the 30 included reports	no additional eligible reports		

Table S3. Methodological characteristics of the cutaneous study units (sources of heterogeneity)

Study unit	Lymphatic marker	Blood vessel marker	Compartments	Quantification	Cut-off	Effect measure	Adjustment
Dadras 2003 [32]	LYVE-1	CD31	Intratumoral, peritumoral	Computer-assisted relative lymphatic vessel area	1.5% relative area	Difference between groups; DFS and OS (Kaplan-Meier)	Matched design (thickness, age, sex)
Straume 2003 [33]	LYVE-1, podoplanin	Not assessed	Intratumoral, peritumoral	Hot-spot counts per mm2	Median	HR (Cox)	Multivariable
Dadras 2005 [34]	LYVE-1	Not assessed	Intratumoral, peritumoral hot-spots	Hot-spot counts	ROC-derived	Sensitivity, specificity for SLN status	Compared with thickness
Yun 2011 [35]	D2-40 with S-100	Not assessed	Regression areas; LI	Counts in regression areas; LI presence	High vs low	HR (Cox) for time to first metastasis and MSS	Multivariable
Shayan 2012 [36]	Podoplanin	Not assessed	Peripheral vs central	Counts per unit area	Continuous	Difference in means; peripheral-to-central ratio	Matched pairs
Toader 2014 [41]	D2-40	CD31	Intratumoral, peritumoral	Hot-spot counts at 200x	None	Correlation	None
Aung 2015 [42]	D2-40; D2-40/S-100 for LVI	VEGFR-2, endocan	Whole tumour	Counts (method not stated)	Median	Association with prognosticators; LVI detection rate	Regression adjusted for MVD and LVD
Pasquali 2015 [43]	D2-40 (LVI, LVD)	CD31 (BVI, BVD)	Intratumoral, peritumoral	Counts	Not stated	Detection rates; HR for survival	Multivariable
Pastushenko 2015 [44]	D2-40 with Ki-67	CD34 with Ki-67	Intratumoral, peritumoral	Chalkley count, MVD, endothelial proliferation fraction (consensus method)	Not stated	OR for SLN, non-SLN, distant metastasis; HR for DFS and OS	Multivariable
Zidlik 2015 [45]	Not assessed	Endothelial marker (MVD)	Central, peripheral	Light microscopy counts	None	Correlation with pT and VGP	None
Spiric 2015 to 2020 [46–50]	D2-40; D2-40/S-100 for LI	Not assessed	Intratumoral, peritumoral	Computer-assisted morphometry (LVD, LVA)	Continuous (per unit) and median	OR for nodal metastasis; HR for DFS and MSS; AUC	Multivariable
Feldmeyer 2016 [51]	D2-40 with or without MITF (LVI)	Not assessed	LVI presence	Detection rate	Presence	Association with SLN status	None (univariable)
Pastushenko 2016 [52]	D2-40 with Ki-67	CD34 with Ki-67	Intratumoral, peritumoral; primary and SLN	Chalkley count, MVD, proliferation rate	Not stated	OR for non-SLN status; HR for OS	Multivariable
Bgatova 2016 [53]	Podoplanin, LYVE-1	CD34	Peritumoral	Volume density	None	Difference between stage groups	None
Moy 2017 [54]	D2-40 with S-100 (LI)	Not assessed	LI presence	Detection rate	Presence	SLN positivity; progression-free survival	None (univariable)
Moy 2019 [55]	D2-40 with S-100 (LI, angiotropism)	CD31	Presence	Detection rate by method	Presence	Association with progression and distant metastasis	Multivariable (non-significant)
Bayram 2021 [56]	D2-40 with S-100 (LVI)	CD31, CD34 with S-100	LVI presence	Detection rate by combination	Presence	OR for SLN status; HR for OS	Multivariable
Straker 2022 [57]	D2-40 with MART-1, SOX-10 or S-100	Not assessed	LI presence	Detection rate	Presence	AUC for lymphatic spread	None
Zheng 2023 [58]	LYVE-1	Not assessed	Intratumoral,	Counts per high-power	None	Difference between groups;	None

			peritumoral	field		correlation with Ang-2	
Femel 2024 [59]	Multiplex panel (lymphatic subtypes)	Multiplex panel	Localisation in and around tumour nests	Quantitative multiplex IHC, digital image analysis	None	Association with regional progression and T cell infiltration	None (exploratory)
Tan 2024 [60]	D2-40 with Ki-67 (multiplex IF)	CD31 with Ki-67	Whole section	Presence of endothelial Ki-67 co-expression	Presence	aHR for MSS and recurrence	Age, sex, Breslow depth, ulceration, site

Reference numbers in square brackets refer to the reference list of the main text. Ang-2, angiopoietin-2; aHR, adjusted hazard ratio; AUC, area under the receiver operating characteristic curve; BVD, blood vessel density; BVI, blood vessel invasion; DFS, disease-free survival; HR, hazard ratio; IF, immunofluorescence; IHC, immunohistochemistry; LI, lymphatic invasion; LVA, lymphatic vessel area; LVD, lymphatic vessel density; LVI, lymphovascular invasion; MSS, melanoma-specific survival; MVD, microvascular density; OR, odds ratio; OS, overall survival; SLN, sentinel lymph node; VGP, vertical growth phase.
